# Clinical efficacy analysis of platelet-rich plasma combined with Xianling Gubao Capsules in the treatment of early-middle stage knee osteoarthritis

**DOI:** 10.3389/fmed.2026.1786275

**Published:** 2026-05-13

**Authors:** Bao-Lin Yang, Zai-Yong Guan, Wen-Long Guo, Shan-Zhen Hou, Wen Gao, Bing Zhou, Ting-Yong Sun

**Affiliations:** Department of Orthopedics, Yangzhou University Affiliated Gaoyou People’s Hospital, Yangzhou, Jiangsu, China

**Keywords:** integrated traditional Chinese and western medicine treatment, knee osteoarthritis, platelet-rich plasma, Smad-1, transforming growth factor-β1, Xianling Gubao Capsules

## Abstract

**Objective:**

To assess the clinical efficacy of platelet-rich plasma (PRP) combined with Xianling Gubao Capsules in the treatment of early to moderate knee osteoarthritis (KOA, Kellgren-Lawrence grade I–III), and to explore exploratory associations with serum levels of transforming growth factor-β1 (TGF-β1) and Smad-1, thereby providing clinical and mechanistic evidence for an integrated traditional Chinese and Western medicine approach in managing early-moderate KOA.

**Methods:**

A total of 60 patients with early to moderate KOA admitted to Gaoyou People’s Hospital between January 2024 and January 2025 were enrolled in this single-center, prospective, randomized controlled trial (RCT) and randomly assigned to a control group (*n* = 30) or a study group (*n* = 30). The control group received intra-articular PRP injection (5 mL per injection, once weekly for three consecutive weeks). The study group received the same PRP regimen plus oral Xianling Gubao Capsules twice daily for 6 weeks. Primary outcome: change in the Western Ontario and McMaster Universities (WOMAC) total score at 6 months after treatment. Secondary outcomes: the visual analog scale (VAS) score, clinical effective rate, serum TGF-β1 and Smad-1 levels, and adverse reactions. Clinical symptoms were evaluated using the VAS and the WOMAC before treatment, 1 month after treatment, and 6 months after treatment. Serum TGF-β1 and Smad-1 levels were measured by enzyme-linked immunosorbent assay (ELISA) at the same time points. Adverse reactions were recorded and compared between the two groups.

**Results:**

All patients completed follow-up. The clinical effective rate was 96.7% in the study group and 90.0% in the control group, with no statistically significant difference (*P* > 0.05). Baseline characteristics were comparable between groups. After treatment, VAS and WOMAC scores significantly decreased in both groups (*P* < 0.05), with lower scores observed in the study group compared to the control group (*P* < 0.05). Serum TGF-β1 levels significantly decreased in both groups after treatment (*P* < 0.05), with a greater reduction in the study group (*P* < 0.05). Although serum Smad-1 levels did not change significantly within each group before and after treatment (*P* > 0.05), the post-treatment level in the study group was higher than that in the control group (*P* < 0.05). The incidence of adverse reactions did not differ significantly between the two groups (*P* > 0.05).

**Conclusion:**

Platelet-rich plasma combined with Xianling Gubao Capsules effectively alleviates pain and improves joint function in patients with early to moderate KOA, with superior efficacy compared to PRP alone. Exploratory serum biomarker findings suggest potential associations with downregulation of TGF-β1 and upregulation of Smad-1, which may be linked to inhibited joint inflammation and promoted cartilage repair. The combined regimen is safe and does not increase the risk of adverse reactions.

## 1 Background

Knee osteoarthritis (KOA) is a prevalent chronic degenerative joint disease among middle-aged and elderly populations, characterized by core pathologies including cartilage degeneration, synovial inflammation, and osteophyte formation (1, 2). The condition leads to knee pain and functional impairment, significantly reducing patients’ quality of life and increasing the healthcare burden (3). As the global population ages, the prevalence of KOA continues to rise, making the exploration of effective interventions for early- to moderate-stage KOA a key focus in orthopedic research.

The primary goals in managing early- to moderate-stage KOA are to alleviate pain, slow cartilage degeneration, and improve joint function. In recent years, the combined use of biological agents and traditional Chinese medicine (TCM) has gained increasing research attention (4–6). Platelet-rich plasma (PRP), an autologous biological preparation rich in growth factors such as platelet-derived growth factor (PDGF) and transforming growth factor-β (TGF-β), promotes chondrocyte proliferation, enhances extracellular matrix synthesis, and modulates local inflammatory responses, thereby supporting cartilage repair. It has been widely adopted in the clinical management of KOA (7, 8).

Xianling Gubao Capsule is a well-established Chinese patent medicine used in the treatment of KOA. Developed based on TCM principles of “tonifying the liver and kidney, strengthening tendons and bones, and promoting blood circulation to remove stasis,” its main herbal components include *Epimedium brevicornu* Maxim., *Dipsacus asper* Wall. ex DC., and *Salvia miltiorrhiza* Bunge. Modern pharmacological studies indicate that this formulation can suppress synovial inflammation, improve joint microcirculation, and downregulate cartilage-degrading enzymes such as matrix metalloproteinases, suggesting potential synergistic benefits when combined with conventional Western therapies for KOA (9, 10). TGF-β1 is a crucial regulator of cartilage metabolism, maintaining homeostasis under physiological conditions, while its dysregulation in KOA may exacerbate inflammation and degenerative processes. Smad-1 is a key downstream signaling molecule involved in cartilage repair, closely associated with regenerative capacity (11, 12). However, clinical evidence is still lacking regarding whether the therapeutic synergy of PRP combined with Xianling Gubao Capsules operates through modulation of serum TGF-β1 and Smad-1 levels, and the underlying mechanism remains unclear.

In this study, we conducted a single-center, prospective, randomized controlled trial (RCT) to compare the efficacy of PRP combined with Xianling Gubao Capsules versus PRP alone in patients with early- to moderate-stage KOA. Serum levels of TGF-β1 and Smad-1 were monitored to evaluate treatment effects. The aim was to clarify the clinical benefits of the combined regimen and preliminarily explore exploratory biomarker associations related to the TGF-β1/Smad-1 pathway, thereby providing an evidence-based rationale for integrated traditional Chinese and Western medicine in the management of early- to moderate-stage KOA.

## 2 Materials and methods

### 2.1 Study design

This study adopted a single-center, prospective, randomized controlled trial (RCT) design with a single-blinding (outcome evaluator and laboratory inspector blinding). Randomization was completed by two independent researchers (statistician and orthopedic attending physician, non-participants in treatment/evaluation) using the random number table method. No placebo capsule was used for the control group; the blinding measures included placebo administration for the control group, anonymous number recording of clinical data, and blinding of evaluators/laboratory inspectors/statisticians to grouping information. The PRP injection surgeon was unblinded due to operational requirements, and no blinding deviation occurred during the study.

### 2.2 Study objects

**FIGURE 1:**
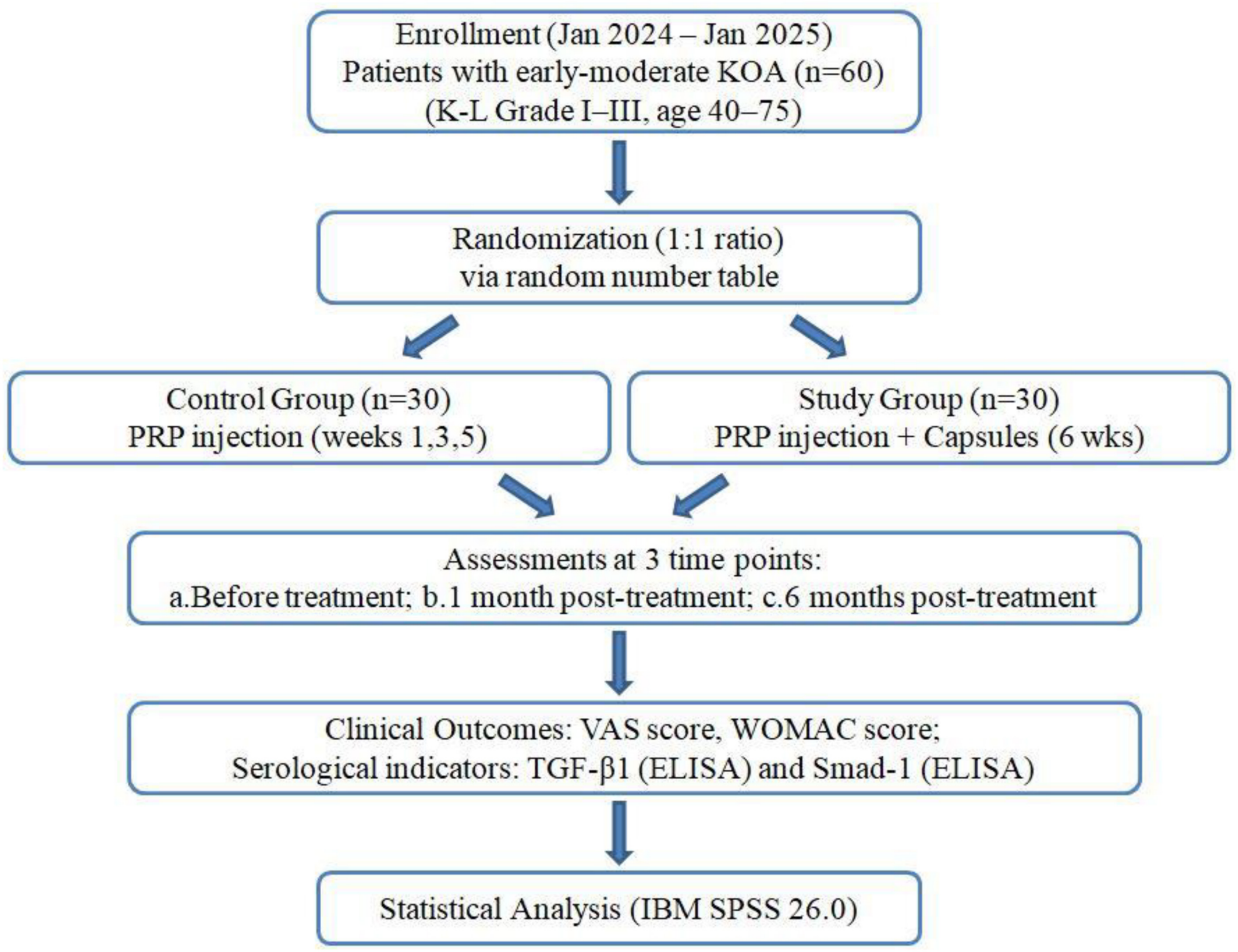
Flow chart showing the study process.

A total of 60 patients diagnosed with early to moderate stage knee osteoarthritis (KOA) at the Orthopedic Outpatient Department of Yangzhou University Affiliated Gaoyou People’s Hospital between January 2024 and January 2025 were enrolled in this study. A detailed flow chart showing the entire study process is presented in Figure 1. Sample size calculation: Based on the primary outcome indicator, the two-sample *t*-test formula (α = 0.05, β = 0.2, *d* = 0.8) was used to calculate the minimum sample size of 26 cases per group; 30 cases per group were enrolled considering a 10% dropout rate. This study was implemented in strict accordance with the Declaration of Helsinki (2022 revision) and obtained the ethical approval of the Ethics Committee of Yangzhou University Affiliated Gaoyou People’s Hospital (Approval No.: KY-2024-09-46). All participants were fully informed of the study details and signed the written informed consent form (including consent for sample collection, index detection and privacy-protected result publication).

#### 2.2.1 Diagnostic criteria

Knee osteoarthritis was diagnosed according to Western medical criteria outlined in Diagnosis and Treatment of Osteoarthritis (1), with the following key diagnostic points: (1) Recurrent knee pain within the past month; (2) Audible or palpable crepitus during knee movement; (3) Morning stiffness lasting ≤30 min; (4) Age ≥ 40 years; (5) Standing X-ray findings showing at least one of the following: joint space narrowing, subchondral sclerosis or cyst formation, or marginal osteophytes. Diagnosis was established if criteria (1) + (2) + (3) + (4), or (1) + (2) + (5), or (1) + (4) + (5) were satisfied.

Traditional Chinese medicine (TCM) syndrome differentiation was performed according to the “bone bi syndrome” criteria described in Criteria for Diagnosis and Therapeutic Effect of TCM Diseases and Syndromes (13). All enrolled cases corresponded to the syndrome pattern of “liver-kidney deficiency with blood stasis obstructing the collaterals.” Diagnosis required at least two main symptoms (knee pain, limited movement, soreness and weakness of the waist and knees) plus at least two secondary symptoms (joint stiffness, aggravation after fatigue, dark tongue or petechiae, deep-thready or choppy pulse).

#### 2.2.2 Inclusion criteria

(1) Meeting the diagnostic criteria for KOA and the traditional Chinese medicine (TCM) syndrome differentiation criteria for “liver-kidney deficiency with blood stasis obstructing the collaterals”; (2) Kellgren-Lawrence (K-L) grade I–III on knee radiography (early to moderate stage) (14); (3) Age between 40 and 75 years; (4) Body mass index (BMI) 18.5∼28 kg/m^2^; (5) No history of PRP injection, intra-articular glucocorticoid administration, or other specific KOA treatments within 1 month prior to enrollment; (6) Provision of signed informed consent.

#### 2.2.3 Exclusion criteria

(1) Advanced KOA with Kellgren-Lawrence (K-L) grade IV, presenting severe joint deformity or complete joint-space narrowing; (2) History of knee trauma, active infection, tumor, or other coexisting joint diseases (e.g., rheumatoid arthritis, gouty arthritis); (3) Coagulation dysfunction, thrombocytopenia (platelet count < 100 × 10^9^/L), or severe anemia; (4) Known allergy to any component of PRP or Xianling Gubao Capsules; (5) Severe cardiac, hepatic, or renal insufficiency; (6) Pregnancy or lactation; (7) Presence of psychiatric disorders or inability to cooperate with follow-up and outcome assessment.

#### 2.2.4 Dropout and exclusion criteria

(1) Development of severe adverse events or complications requiring treatment discontinuation during the intervention or follow-up period; (2) Non-adherence to the prescribed regimen, such as missed PRP injections or consecutive medication omissions exceeding 24 h; (3) Initiation of other KOA-targeted therapies outside the study protocol during follow-up; (4) Loss to follow-up or voluntary withdrawal from the study.

### 2.3 Treatment methods

Sixty eligible participants were randomly assigned in a 1:1 ratio to either the control group or the study group (*n* = 30 each) using a random number table. Throughout the treatment period, all patients received standardized health education, which included advice on avoiding activities that increase knee-joint loading (e.g., prolonged walking, stair climbing, and deep squatting) and encouragement to perform regular muscle-strengthening exercises, such as straight-leg raises and isometric quadriceps contractions.

#### 2.3.1 Control group

Patients in this group received intra-articular autologous PRP injections as the sole intervention. PRP was prepared using a double-centrifugation protocol: 30 mL of peripheral venous blood was collected aseptically from the median cubital vein into a citrate-anticoagulated tube. A dedicated medical centrifuge (WG-FZLX-1, Shandong Weigao Xinsheng Medical Devices Co., Ltd.) was used for processing. The first centrifugation was performed at 1500 rpm for 10 min to separate the platelet-rich plasma (upper layer) from red blood cells (lower layer). The plasma layer was carefully aspirated and transferred to a fresh tube, followed by a second centrifugation at 3000 rpm for 15 min. After discarding the upper platelet-poor plasma, approximately 5 mL of PRP was collected from the bottom of the tube, with platelet concentration ≥ 5 × 10^11^/L (230∼280 times the baseline peripheral blood concentration), mean platelet volume (MPV) 10.2 ± 1.5 fL, WBC 2.5 ± 0.8 × 10^9^/L, RBC < 1 × 10^12^/L.

For the injection procedure, the patient was placed in a supine position with the knee flexed at 90°. Following routine skin disinfection and draping, 2% lidocaine hydrochloride injection (1∼2 mL per patient) was used for subcutaneous and periarticular infiltration anesthesia at the superolateral border of the patella; PRP injection was performed 3∼5 min after anesthesia. A needle was inserted from the superolateral border of the patella and 5 mL of PRP was slowly injected. All injection operations were performed by the same senior orthopedic surgeon. After injection, the patient was instructed to perform gentle knee flexion and extension for 5–10 min. Injections were administered once weekly on weeks 1, 3, and 5 (4 weeks after the last non-study KOA treatment) for a total of three injections over a 6-weeks treatment cycle. All enrolled patients had no acute knee injury history within 3 months before enrollment, and the injection timing was not associated with acute injury.

#### 2.3.2 Study group

Patients in this group received a combined regimen consisting of PRP injection plus oral Xianling Gubao Capsules. The preparation, injection technique, dose, and course of PRP administration were identical to those in the control group. Additionally, oral Xianling Gubao Capsules (Guizhou Tongjitang Pharmaceutical Co., Ltd.; National Medical Products Administration Approval No. Z20025337; 0.5 g per capsule) were administered at a dosage of 3 capsules twice daily with warm water for 6 consecutive weeks.

### 2.4 Observation indicators and detection methods

#### 2.4.1 Primary outcome

Change in Western Ontario and McMaster Universities (WOMAC) Osteoarthritis Index total score at 6 months after treatment (core patient-reported outcome for KOA, recommended by international guidelines).

#### 2.4.2 Secondary outcomes

1) Visual Analogue Scale (VAS) pain score at 1 month and 6 months after treatment;
2) Clinical effective rate at 6 months after treatment;
3) Serum TGF-β1 and Smad-1 levels at 1 month after treatment;
4) Incidence of adverse reactions during treatment and follow-up.

#### 2.4.3 Collection of general data

Before initiating treatment, baseline demographic and clinical characteristics–including sex, age, disease duration, BMI, platelet count and K-L radiographic grade–were systematically recorded for both groups to evaluate the comparability of baseline profiles. This step ensured that any observed differences in outcomes could be attributed to the interventions rather than to pre-existing imbalances between the groups.

#### 2.4.4 Clinical efficacy evaluation

Patients in both groups were evaluated at three predefined time points by two fixed orthopedic residents (non-participants in treatment, blinded to grouping): before treatment, 1 month after treatment completion, and 6 months after treatment completion, using the following standardized assessment tools: (1) Visual Analogue Scale (VAS) (15): patients indicated their subjective pain intensity by marking a point on a 10-cm horizontal line, where 0 cm represented “no pain” and 10 cm represented “the worst pain imaginable.” Higher scores reflected greater pain severity. (2) Validated Chinese version of the Western Ontario and McMaster Universities Osteoarthritis Index (WOMAC 3.0) (16, 17): this multidimensional instrument assesses knee osteoarthritis in three domains: pain (5 items, score range 0–20), stiffness (2 items, score range 0–8), and physical function (17 items, score range 0–68). The total score ranges from 0 to 96, with higher scores indicating more severe symptoms and greater functional impairment.

#### 2.4.5 Detection of serological indicators

Fasting peripheral venous blood samples (5 mL) were collected from all patients before treatment and 1 month after treatment completion. Serum was separated by centrifugation, and the concentrations of TGF-β1 and Smad-1 proteins were measured using enzyme-linked immunosorbent assay (ELISA) by three clinical laboratory technicians blinded to patient grouping. Commercial ELISA kits (Shanghai Enzyme-Linked Biotechnology Co., Ltd.) were used, and all procedures were carried out in strict accordance with the manufacturer’s instructions.

#### 2.4.6 Safety evaluation

Throughout the entire treatment period and during the 1-month follow-up after treatment completion, adverse reactions in both groups were closely monitored. These included local injection-site reactions (e.g., redness, swelling, pain, or rash) and systemic reactions (e.g., nausea, vomiting). All observed or voluntarily reported adverse events were documented in detail, including time of onset, severity, potential relationship to the intervention, and final outcome.

### 2.5 Efficacy criteria

Treatment efficacy was assessed according to the Clinical Practice Guidelines for Osteoarthritis (2021 Edition) (1), using the following criteria: (1) Markedly effective: VAS score decreased by ≥70% or WOMAC score decreased by ≥60% after treatment, with significant pain relief, essentially normal joint movement, and no restriction in daily activities. (2) Effective: VAS score decreased by 30%–69% or WOMAC score decreased by 30%–59%, accompanied by partial pain relief, improved joint mobility, and minimal restriction in daily life. (3) Ineffective: VAS score decreased by <30% or WOMAC score decreased by <30%, with no pain improvement or even worsening symptoms, and no improvement in joint function. The clinical effective rate was calculated as follows: Clinical effective rate = (number of markedly effective cases + number of effective cases)/total number of cases × 100%.

### 2.6 Statistical methods

Statistical analysis was performed using IBM SPSS version 26.0. Normally distributed continuous variables are expressed as mean ± standard deviation (x̄ ± SD). Paired *t*-tests were used for within-group comparisons before and after treatment, while independent-samples *t*-tests were applied for between-group comparisons. Categorical variables are reported as number (percentage), and compared using the χ^2^ test. Ordinal data, such as efficacy grades, were analyzed with the Mann-Whitney U test. A *P*-value < 0.05 was considered statistically significant.

## 3 Results

### 3.1 Comparison of clinical data between the two groups

All 60 enrolled subjects completed the full treatment and follow-up period without any dropouts. Baseline characteristics, including sex, age, disease duration, BMI, platelet count and K-L grade, did not differ significantly between the two groups (all *P* > 0.05), confirming balanced and comparable baseline profiles (Table 1).

**TABLE 1:** Comparison of baseline data between the two groups [*n* (%) or x ± s].

Indicators	Control group (*n* = 30)	Study group (*n* = 30)	χ ^2^/*t*-value	*P*-value
Gender (Male/Female)	11/19	13/17	0.278	0.598
Age (years)	62.3 ± 5.8	63.7 ± 6.2	0.903	0.370
Course of disease (years)	3.2 ± 1.5	3.8 ± 1.3	1.656	0.103
BMI (kg/m^2^)	24.8 ± 2.5	25.1 ± 2.3	0.484	0.630
Baseline platelet count (×10^9^/L)	215.6 ± 32.8	220.3 ± 35.2	0.535	0.595
K-L classification (Grade I/II/III)	8/15/7	7/16/7	0.099	0.952

### 3.2 Comparison of clinical efficacy between the two groups

At the 6-months follow-up, the clinical effective rate was 96.7% in the study group (29/30; 18 markedly effective, 11 effective, 1 ineffective) and 90.0% in the control group (27/30; 13 markedly effective, 14 effective, 3 ineffective). No statistically significant difference was observed between the two groups (χ^2^ = 1.071, *P* = 0.301) (Table 2).

**TABLE 2:** Comparison of clinical efficacy between the two groups [*n* (%)].

Groups	Number of cases	Markedly effective	Effective	Ineffective	Effective rate
Control group	30	13 (43.3)	14 (46.7)	3 (10.0)	90.0
Study group	30	18 (60.0)	11 (36.7)	1 (3.3)	96.7
χ^2^ value	–	–	–	–	1.071
*P*-value	–	–	–	–	0.301

### 3.3 Comparison of VAS scores and WOMAC scores between the two groups before and after treatment

Before treatment, no significant differences in VAS or WOMAC scores were observed between the two groups (both *P* > 0.05). At 1 month and 6 months after treatment completion, both groups showed significant reductions in VAS and WOMAC scores compared with baseline (*P* < 0.05). Moreover, the study group exhibited significantly lower scores than the control group at both follow-up time points (all *P* < 0.05) (Tables 3, 4).

**TABLE 3:** Comparison of VAS scores between the two groups before and after treatment (points, x ± s).

Groups	Number of cases	Before treatment	1 month after treatment	6 months after treatment
Control group	30	6.8 ± 1.1	3.5 ± 0.9	2.8 ± 0.7
Study group	30	6.9 ± 1.5	2.9 ± 0.6	2.0 ± 0.4
*t*-value (before treatment)	–	0.294	–	–
*P*-value (before treatment)	–	0.769	–	–
*t*-value (1 month after treatment)	–	–	3.038	–
*P*-value (1 month after treatment)	–	–	0.004	–
*t*-value (6 months after treatment)	–	–	–	5.435
*P*-value (6 months after treatment)	–	–	–	0.000

**TABLE 4:** Comparison of WOMAC scores between the two groups before and after treatment (points, x ± s).

Groups	Number of cases	Before treatment	1 month after treatment	6 months after treatment
Control group	30	58.6 ± 10.2	32.4 ± 8.5	26.3 ± 7.1
Study group	30	59.2 ± 9.8	29.1 ± 7.6	21.8 ± 6.3
*t*-value (before treatment)	–	0.232	–	–
*P*-value (before treatment)	–	0.817	–	–
*t*-value (1 month after treatment)	–	–	2.546	–
*P*-value (1 month after treatment)	–	–	0.014	–
*t*-value (6 months after treatment)	–	–	–	2.597
*P*-value (6 months after treatment)	–	–	–	0.012

### 3.4 Comparison of serum TGF-β1 and Smad-1 levels between the two groups before and after treatment

Before treatment, serum levels of TGF-β1 and Smad-1 did not differ significantly between the two groups (both *P* > 0.05). One month after treatment, TGF-β1 levels decreased significantly in both groups compared with baseline (*P* < 0.05), with a more pronounced reduction in the study group than in the control group (*P* < 0.05). In contrast, Smad-1 levels did not change significantly within either group relative to pre-treatment values (*P* > 0.05). However, the study group showed significantly higher post-treatment Smad-1 levels compared with the control group (*P* < 0.05) (Table 5).

**TABLE 5:** Comparison of serum TGF-β1 and Smad-1 levels between the two groups before and after treatment (pg/ml, x ± s).

Indicators	Groups	Number of cases	Before treatment	1 month after treatment
TGF-β1	Control group	30	125.6 ± 20.3	86.4 ± 15.7
	Study group	30	128.2 ± 19.8	65.3 ± 14.2
*t*-value (before treatment)	–	–	1.004	–
*P*-value (before treatment)	–	–	0.319	–
*t*-value (1 month after treatment)	–	–	–	5.783
*P*-value (1 month after treatment)	–	–	–	0.000
Smad-1	Control group	30	90.2 ± 8.6	93.6 ± 9.1
	Study group	30	92.2 ± 8.3	99.4 ± 8.7
*t*-value (before treatment)	–	–	0.917	–
*P*-value (before treatment)	–	–	0.363	–
*t*-value (1 month after treatment)	–	–	–	2.523
*P*-value (1 month after treatment)	–	–	–	0.014

### 3.5 Safety comparison between the two groups

During the treatment period and the 1-month follow-up, mild adverse events were reported in 3 of 30 patients (10.0%) in the control group (injection-site pain in 2 cases and rash in 1 case) and in 2 of 30 patients (6.7%) in the study group (injection-site pain in 1 case and nausea in 1 case). All events were mild and resolved with symptomatic management such as local cold compress or temporary drug withdrawal. No serious adverse events, such as severe allergic reactions or liver dysfunction, occurred. The incidence of adverse reactions did not differ significantly between the two groups (χ^2^ = 0.218, *P* = 0.064).

## 4 Discussion

The pathophysiology of knee osteoarthritis (KOA) involves complex interactions among multiple factors, with core pathological changes including chondrocyte apoptosis, abnormal extracellular matrix degradation, chronic synovitis, and osteophyte formation. Within this process, the TGF-β/Smad signaling pathway plays a central role in regulating cartilage metabolism and repair (18). TGF-β1, a key upstream mediator of this pathway, promotes chondrocyte proliferation and type II collagen synthesis under physiological conditions by activating downstream effectors such as Smad-1 and Smad-2, thereby maintaining cartilage homeostasis. However, under KOA pathological conditions, TGF-β1 is often overexpressed, stimulating synovial fibroblasts to secrete pro-inflammatory cytokines (e.g., IL-1β, TNF-α) and up-regulate matrix metalloproteinase (MMP) activity, which accelerates cartilage degeneration and synovial inflammation (19, 20). Smad-1, a critical downstream signaling molecule of TGF-β1, mediates transcriptional activation of genes involved in cartilage repair. Studies indicate that down-regulation of Smad-1 may impair intrinsic cartilage repair capacity and contribute to KOA progression (21).

In the present study, the primary outcome (WOMAC total score at 6 months) confirmed significantly better functional improvement in the study group than in the control group. Although the total clinical effective rate showed no statistical difference, the secondary outcomes (VAS and WOMAC scores) consistently supported the superiority of the combined regimen.

At the biomarker level, serum TGF-β1 levels decreased significantly after treatment in both groups, with a more pronounced reduction in the study group. Although serum Smad-1 levels did not change significantly within either group before versus after treatment, post-treatment Smad-1 concentrations were significantly higher in the study group than in the control group. These findings represent exploratory associations with the TGF-β/Smad signaling pathway, rather than a confirmed mechanistic pathway, and suggest that the combination of PRP and Xianling Gubao Capsules may modulate related serum biomarkers to potentially inhibit local joint inflammation and promote cartilage repair, consistent with previous reports (22, 23).

Mechanistically, PRP exerts its effects through concentrated bioactive growth factors: platelet-derived growth factor (PDGF) directly stimulates chondrocyte proliferation; transforming growth factor-β (TGF-β) promotes extracellular matrix synthesis at physiological concentrations; and epidermal growth factor inhibits chondrocyte apoptosis (8). PRP also alleviates chronic synovitis by modulating synovial macrophage activity and suppressing pro-inflammatory cytokine release (24). Xianling Gubao Capsules, formulated based on traditional Chinese medicine principles of “tonifying the liver and kidney, strengthening tendons and bones, and promoting blood circulation to remove stasis,” exhibit multi-pathway pharmacological actions. Its active components have been shown to: (1) regulate the hypothalamic-pituitary-adrenal axis and improve the chondrocyte metabolic microenvironment, reducing apoptosis (e.g., icariin) (25); (2) inhibit platelet aggregation, improve joint microcirculation, and relieve synovial congestion and edema (e.g., tanshinone) (26); and (3) suppress MMP expression, down-regulate inflammatory cytokines such as IL-6 and TNF-α, and protect cartilage matrix (e.g., *Dipsacus asper* saponins) (27).

The combined regimen exemplifies the complementary advantages of integrated traditional Chinese and Western medicine. PRP, delivered via intra-articular injection, provides localized, rapid anti-inflammatory and reparative effects. Xianling Gubao Capsules, administered orally, support systemic regulation and optimize the articular cartilage microenvironment. Notably, the two modalities appear to act synergistically on the TGF-β/Smad pathway: PRP may directly modulate local TGF-β1 levels, while the multi-target effects of Xianling Gubao Capsules up-regulate Smad-1 expression, thereby enhancing pro-repair signaling. This integrated approach–combining targeted local intervention with systemic modulation, and acute action with sustained regulation–likely underlies the observed clinical synergy.

Regarding safety, the overall incidence of adverse reactions did not differ significantly between groups. All reported events were mild and resolved promptly with symptomatic measures (e.g., local cold compress for injection-site pain, temporary drug withdrawal for gastrointestinal symptoms). These results indicate that the combination of PRP and Xianling Gubao Capsules has a favorable safety profile in early- to moderate-stage KOA, without increasing additional risk, thereby supporting its further clinical application.

## 5 Limitations and future directions

This study has several limitations. First, the sample size was relatively small (*n* = 60) and recruitment was limited to a single center, which may introduce selection bias and restrict the generalizability of the findings. Second, the follow-up duration was relatively short (up to 6 months), which limits evaluation of the regimen’s long-term efficacy and its potential to delay disease progression. Third, Xianling Gubao Capsules-only group was not included, making it impossible to distinguish the independent therapeutic effect of the capsules and the synergistic effect of the combination therapy. Fourth, this study only assessed serum levels of TGF-β1 and Smad-1, without histopathological evaluation of articular cartilage; the mechanistic interpretations are based on exploratory serum biomarker associations and not direct causal evidence.

Future research should aim to expand sample sizes, incorporate multiple centers, and extend follow-up periods. The core improvement direction is to set up three parallel groups (PRP alone, Xianling Gubao Capsules alone, PRP + Xianling Gubao Capsules) in large-sample studies, to clarify the independent therapeutic effect of each method and the synergistic effect of the combination. In addition, complementary approaches such as detection of serum/synovial fluid inflammatory factors (IL-6, TNF-α, MMPs), quantitative magnetic resonance imaging (MRI) of cartilage, and histopathological examination of articular tissues should be adopted to further verify the underlying mechanism of the combined regimen.

## 6 Conclusion

In summary, the combination of platelet-rich plasma (PRP) and Xianling Gubao Capsules demonstrates clear clinical benefits in patients with early- to moderate-stage knee osteoarthritis (KOA), effectively relieving knee pain and improving joint function. Exploratory serum biomarker analysis shows associations with downregulated TGF-β1 and upregulated Smad-1, which may be related to anti-inflammatory and cartilage repair effects. Based on its favorable efficacy and safety profile, this combined regimen represents a promising therapeutic option worthy of broader clinical adoption in the management of early- to moderate-stage KOA.

## Data Availability

The original contributions presented in this study are included in this article/supplementary material, further inquiries can be directed to the corresponding author.
